# Statistical analysis in support of maintaining a healthy traditional Siamese cat population

**DOI:** 10.1186/s12711-020-00596-w

**Published:** 2021-01-06

**Authors:** Arthur M. A. Pistorius, Ineke Blokker

**Affiliations:** 1 260 Centre for Molecular and Biomolecular Informatics, Radboud University Medical Centre, PO Box 9101, NL-6500 HB Nijmegen, The Netherlands; 2 Workgroup Traditional Siamese Cat Breeders in The Netherlands (WTSN), NL-1815 HC Alkmaar, The Netherlands

## Abstract

**Background:**

For many years, breeders of companion animals have applied inbreeding or line breeding to transfer desirable genetic traits from parents to their offspring. Simultaneously, this resulted in a considerable spread of hereditary diseases and phenomena associated with inbreeding depression.

**Results:**

Our cluster analysis of kinship and inbreeding coefficients suggests that the Thai or traditional Siamese cat could be considered as a subpopulation of the Siamese cat, which shares common ancestors, although they are considered as separate breeds. In addition, model-based cluster analysis could detect regional differences between Thai subpopulations. We show that by applying optimal contribution selection and simultaneously limiting the contributions by other breeds, the genetic diversity within subpopulations can be improved.

**Conclusion:**

In principle, the European mainland Thai cat population can achieve a genetic diversity of about 26 founder genome equivalents, a value that could potentially sustain a genetically diverse population. However, reaching such a target will be difficult in the absence of a supervised breeding program. Suboptimal solutions can be obtained by minimisation of kinships within regional subpopulations. Exchanging animals between different regions on a small scale might be already quite useful to reduce the kinship, by achieving a potential diversity of 23 founder genome equivalents. However, contributions by other breeds should be minimised to preserve the original Siamese gene pool.

## Background

Worldwide, there is growing concern about the loss of biodiversity, which is caused by the ever-increasing human population. The quantitative reduction of biotopes and over-exploitation of wild species result in the fragmentation of populations and reduction in their sizes which, in turn, increase the degree of inbreeding. Finally, inbreeding may result in inbreeding depression, which manifests itself by reduced reproductive fitness and reduced ability to adapt to changing environmental conditions. All these factors negatively influence the possibility to restore the size of populations to a reasonable level. Thus, the risk of extinction increases [1].

Closer to home, this also applies to the breeding of domestic animals and companion animals. For many years, inbreeding has been an accepted method to transfer desirable genetic traits to the offspring in a predictable way, thereby increasing the economic or aesthetic value of the animals. However, this approach that deliberately reduces the heterozygosity level, simultaneously increases the risk of combining deleterious genes, and thus results in increasing the prevalence of several hereditary diseases or inbreeding depression which negatively influence the health of the animals and the population at large.

The Siamese cat is a very ancient breed that originated from the Kingdom of Siam (nowadays Thailand), and was already known in the 14th century [2]. The breed was imported into the United Kingdom in the late 19th century and then spread to other European countries. Starting in the mid-20th century, Siamese cats that exhibited a very slender body and wedge-shaped head conformation became fashionable. The classical shaped cats were not registered or accepted for shows anymore, which almost led to extinction of the original type in Europe.

A few breeders started to re-introduce the classical type and, in 1995, the traditional Siamese was once again accepted by some registries. The new population was built on founders that were imported from several countries around the world, and on selected Siamese cats of European origin, which had a less extreme conformation. The registered breed name Siamese remained connected to the modern type and from that time on, the classical type became known as the Thai cat, but this definition is not accepted worldwide. In the United States of America, the most important registry, the Cat Fanciers’ Association (CFA), does not recognise the Thai as a breed [3] whereas The International Cat Association (TICA) defines the Thai as a contemporary cat, which has proven to be directly imported from Thailand [4].

Meanwhile, heavy selection pressure and misfortunate outcrosses with other breeds resulted in an increased prevalence of several hereditary diseases within the contemporary Siamese cat population, e.g. progressive retina atrophy (PRA) [5] and traits which are associated with inbreeding depression such as skeletal deformations and a reduced life span. In contrast, up to date, the Thai cat population is essentially free from the hereditary diseases that have been detected within the contemporary Siamese breed except for isolated cases. Thus, the Thai and the Siamese cat populations appear to have developed differently.

In this article, we report on the historic development and present status of the genetic diversity within the Siamese and Thai cat breeds that are present on the European mainland. We carried out a cluster analysis to reveal the relationships between both breeds and to explore regional differences in the distribution of their genetic diversity. We also calculated the mean kinship of breeding animals per cluster, which provides a more useful parameter to maintain or improve genetic diversity within the current Thai breed. Our aim is to preserve a healthy cat population without any exaggeration in appearance and that is related to the original Siamese cats that were present in Europe until about 1970.

## Methods

### Data

For this study, pedigree information was obtained from associated breeders, registries and on-line databases [6, 7] especially in the Netherlands, Belgium, Germany and the United Kingdom (Additional file 1). The database contains 39576 entries among which 4998 Thai (EMS code [8]: THA), 28770 Siamese (EMS code: SIA), 4446 Oriental Shorthairs (EMS code: OSH) and 737 Balinese (EMS code: BAL) cats, excluding unspecified variants of these breeds. The remaining 625 entries are from 25 other breeds, which were used shortly after the Second World War to rebuild the feline livestock and to create new breeds such as the Oriental Shorthair [2]. After 2000, active data collection on non-Thai breeds was stopped, except for animals which were related to the Thai, e.g. in the case of occasional outcrossing with Siamese cats.

The data collection is known to be incomplete for the periods after the Second World War, when the registries had to be rebuilt, and towards the end of the 20th century when the traditional Siamese cats were not accepted anymore for registration or for entering shows, since they did not meet the new breed standards. The percentage of unknown parentage is about 9 %.

The database of the CFA Siamese Breed Council [6] lists 49 animals that were imported from Thailand, Hong Kong or India in the early 20th century and can be regarded as the founders of the European Siamese and Thai population. The most distant founder was traced more than 50 generations back. The average equivalent complete generations of the current Thai cat population is 12.3. After 20 generations, the percentage of pedigree completeness is 21 % (Additional file 2).

Since subsequent generations show a strong overlap and full demographic details are not available, throughout this work we define a population as the parents of the kittens, born in two consecutive years.

### Definitions

In this work, we base our results on the concept of mean kinship as developed by Ballou [9] and Lacy [10]. The mean kinship is the mean of all pairwise kinship coefficients between an individual and all other breeding animals, including itself, according to:

1 $$\begin{aligned} mk_{i} = \frac{1}{N} \sum _{j=1}^{N} f_{ij}. \end{aligned}$$

The individual pairwise kinship coefficients, $f_{ij}$, are derived from the pedigrees, using the tabular method [11]. The average mean kinship (${\overline{mk}}$) or population mean kinship is the average of all mean kinship values over the entire population, *J*, under study:

2 $$\begin{aligned} \overline{mk}(J) = \frac{1}{N^{2}} \sum _{i=1}^{N} \sum _{j=1}^{N} f_{ij}, \end{aligned}$$

and represents the probability that two randomly chosen alleles from the population *J* are identical-by-descent. From this parameter, gene diversity (GD) is calculated as

3 $$\begin{aligned} GD(J) = 1 - \overline{mk}(J). \end{aligned}$$

In addition, in the same way, Wellmann [12] defined the conditional gene diversity *condGD*(*J*) of a birth cohort *J* as the probability that two alleles randomly chosen from the population are not identical-by-descent, under the condition that both alleles descend from native founders. When conservation of the original gene pool is an objective of a breeding program, this definition is a more adequate descriptor of the gene diversity within a population, which contains contributions from other breeds. In this situation, the aim is to maximise the conditional gene diversity while simultaneously limiting the genetic contribution by other breeds.

Alternatively, genetic diversity can be described in terms of founder genome equivalents or *FGE*. This is the minimum number of unrelated founders required to create a population having the same genetic diversity as the currently investigated population. The *FGE* is approximated by:

4 $$\begin{aligned} FGE(J) = \frac{1}{2\overline{mk}(J)} = \frac{1}{2(1-GD(J))}. \end{aligned}$$

Similarly, the native genome equivalent, *NGE*(*J*) can be defined as the minimum number of unrelated native founders required to create a population having the same conditional gene diversity as the currently investigated population [12]:

5 $$\begin{aligned} NGE(J) = \frac{1}{2(1-condGD(J))}. \end{aligned}$$

Potential diversity is the maximum genetic diversity that can be obtained when minimising the population mean kinship by applying Optimal Contribution Selection (OCS):

6 $$\begin{aligned} GD(J)_{pot} = 1 - \overline{mk}_{min}. \end{aligned}$$

A vector, $\mathbf {c_{oc}}$, that contains the contributions of the breeding animals in population *J*, has to be solved to minimise the population mean kinship in the next generation [13]:

7 $$\begin{aligned} \overline{mk}_{min} = \mathbf {c'_{oc}Mc_{oc}}. \end{aligned}$$

In this equation, $\mathbf {M}$ is the kinship matrix, including the kinship coefficients of breeding animals with themselves. $\mathbf {c_{oc}}$ can be solved by expanding to a Lagrange equation and subsequently determining where the derivative to $\mathbf {c}$ equals zero. Various algorithms exist to obtain $\mathbf {c_{oc}}$. In this work, serial least squares quadratic programming (SQP) [14] was used to determine optimal solutions for $\mathbf {c_{oc}}$.

As for *FGE*, potential diversity, $N_{oc}(J)$, can be expressed in founder genome equivalents:

8 $$\begin{aligned} N_{oc}(J) = \frac{1}{2\overline{mk}_{min}}. \end{aligned}$$

The inbreeding coefficient is the probability that an individual receives the same allele from each parent. Average inbreeding (${\overline{F}}$) is the mean of the coefficient of inbreeding of all breeding individuals and is an indicator of the risk of inbreeding depression. Individual inbreeding coefficients, $F_{i}$, can be calculated from the main diagonal of the kinship matrix by doubling the diagonal elements and subsequently subtracting 1 from the results:

9 $$\begin{aligned} F_{i} = 2*f_{ii} - 1. \end{aligned}$$

The average coefficient of inbreeding thus becomes:

10 $$\begin{aligned} \overline{F} = \frac{1}{N} \sum _{i=1}^{N} (2*f_{ii} - 1). \end{aligned}$$

### Data analysis

Statistical analysis was carried out using the R the statistics program version 3.5.3 [15] including the optiSel version 2.0.2 package, developed by Wellmann [16, 17]. This package contains most of the functions necessary to calculate kinship matrices, breed contributions and minimisation functions, which are needed for OCS.

Prior to further analysis, individuals that are not founders but for which parent information was missing were excluded by setting a threshold year, after which the breed name of these animals is set to Unknown. The parameter “lastNative=1940” was used for the Siamese and “lastNative=2005” for the Thai. Furthermore, individuals with less than 3 equivalent complete generations were excluded.

We investigated two OCS scenarios, i.e. by using the “min.pKin” objective function to minimise the kinship in the next generation and the “min.pKinatN” objective function while simultaneously restricting the contribution by other breeds, which minimises the kinship at native alleles. In principle, the latter approach can be used to reduce the influence of other breeds to next generations of the breed under investigation. Minimisation was performed, using the sequential least-squares quadratic programming (SQP) algorithm, which is available through the nloptr package [14], by setting the opticont solver parameter to “slsqp”. This follows the recommendation by Wellmann [16, 17] for maximising genetic diversity at native alleles. Other algorithms failed to reach a minimum in several cases. Depending on the chosen scenario, additional boundary conditions were applied:

- Optimise both male and female contributions
- Limit the increase in inbreeding by setting an upper boundary to the mean kinship according to the equation $ub.mk=\overline{mk} + (1-\overline{mk})*\Delta F$ with the rate of increase in inbreeding: $\Delta F = 1/(2 N_{e} L)$ [18]. The effective population size, ${N_{e}}$, was approximated by: $N_{e} = 4*N_{male}*N_{female}/(N_{male} + N_{female})$. The generation interval was estimated as $L = 3.2$ years
- Limit the contribution by other breeds by setting a lower boundary to the contribution by the native breed. The contribution by native individuals should be larger as compared to the current population.

To identify subpopulations, we performed a hierarchical cluster analysis of the current breeding animals, which were the parents of the kittens, born in two consecutive years. The input datasets consisted of 200 Thai (from the period 2016–2017) or 462 Siamese individuals (from the period 1998-1999). The function “hclust” was applied, in which the unweighted pair group (UPGMA) method [19, 20] is implemented by using the “average” method. The individual mean kinship values obtained according to Eq. (1) and inbreeding coefficients obtained according to Eq. (9) were used as input data. Since both parameters range between 0 and 1, no input scaling was applied. The optimal number of clusters was estimated using the cubic clustering criterion, the pseudo-$t^{2}$ criterion and the pseudo-F criterion as implemented in the NbClust package [21].

Alternatively, we explored the use of a model-based cluster analysis applied to the same input data [22, 23]. In this approach, each cluster is represented by a normal distribution, which is characterised by a mean vector for mean kinship, a mean vector for inbreeding coefficients, a covariance matrix and a probability that a particular observation belongs to the assigned cluster. The mean vector has as many elements as the number of clusters and contains the centre of the cluster in both dimensions (i.e. mean kinship and inbreeding coefficient). The covariance matrix determines the appearance of the cluster in terms of volume, shape and orientation. Model options for each parameter are variable (V), equal (E) or indexed (I). Subsequently, the model parameters are optimised for a variable number of input clusters using a maximum likelyhood algorithm. The optimal number of clusters is selected using the Bayesian information criterion (BIC), which should have a maximal value.

Clustering solutions were visualised using the “clusplot” function. This function performs a principal component analysis (PCA) on the input data and uses the subdivision into groups as second input to identify the clusters by drawing ellipses around the data points (supervised learning).

Preliminary results on smaller datasets were validated using the PMx software version 1.3.20150713 [24].

## Results

### History of the Thai cat population

**Fig. 1:**
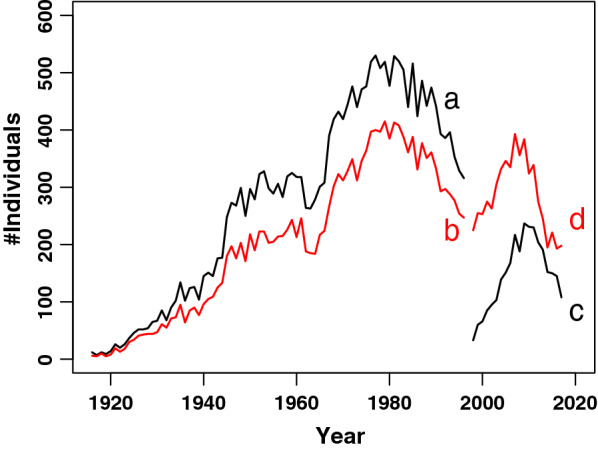
Demographic evolution of the Siamese and Thai breed.** a** Number of unique Siamese breeding individuals;** b** Number of Siamese kittens born (red);** c** Number of unique Thai breeding individuals;** d** Number of Thai kittens born (red). At the time of this study, no data were available for the Siamese breed after 2000

The growth of the population of Siamese cats is evident from the number of breeding individuals until 1995 (Fig. 1). After the Thai cat was accepted as a separate breed, new entries were recorded as Thai and only Siamese cats which were still related to the current Thai population were recorded. The number of Thai breeding individuals peaked in 2011 but then declined to 101 unique individuals in 2017. This decline requires attention but the number of breeding individuals is still sufficiently large to maintain a viable population. In the Netherlands between 2000 and 2015, 433 litters were born, producing 1660 Thai kittens. The current average litter size is 4.2. Only a few cats become breeding candidates. Since we do not receive full information from breeders abroad, the number of newborn Thai kittens is underestimated. The number of kittens within the Siamese breed is even smaller than the number of parents, partly because in the past, pedigrees were only issued for breeding candidates.

**Fig. 2:**
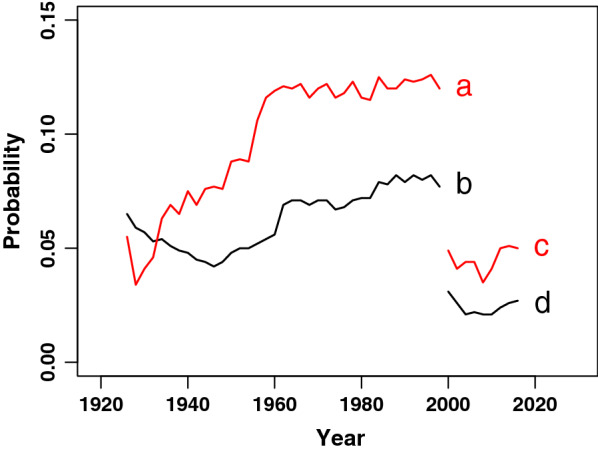
Historic development of inbreeding and mean kinship.** a** Historic development of coefficient of inbreeding within the Siamese breed (SIA);** b** Population mean kinship (SIA);** c** Coefficient of inbreeding within the Thai breed (THA);** d** Population mean kinship (THA) in The Netherlands and surrounding countries. A period of 2 years was taken as an approximate generation interval. At the time of this study, no data were available for the Siamese breed after 2000

Figure 2 shows the historic development of the average coefficient of inbreeding and the population mean kinship of the Siamese (SIA. Trace a and b) until 1999 since, from the perspective of the traditional Siamese breeders, there was no need to collect pedigree data for modern Siamese cats anymore (Additional file 3). The historic development of the Thai (THA) breed, leading to the current population up to 2017 is shown in Trace c and d. Since subsequent generations show a strong overlap, the mean kinship and coefficient of inbreeding are calculated for the parents of each birth cohort over two subsequent years. The dataset consisted of individuals from Belgium, Germany and the Netherlands, with historic additions from the United Kingdom and more recent additions from Eastern European countries. In the early 20th century, strong fluctuations appeared during the period when the Siamese breed was established in Europe. Around 1930, the inbreeding coefficient surpassed the population mean kinship. After a small decrease during the Second World War, inbreeding and population mean kinship rose steadily with an additional jump around 1960. During this period, the modern Siamese type and the Oriental Shorthair (OSH) were developed [2]. The OSH breed has a body shape similar to that of the modern Siamese but it is characterised by a solid colored, non-pointed coat and green eyes. The high inbreeding coefficient relative to the population mean kinship indicates a population structure that results from non-random mating. For the Siamese breed, a population mean kinship of 0.08 and an average inbreeding coefficient of 0.12 were obtained at the end of the data collection in 1998-1999. The Oriental Shorthair showed a population mean kinship of 0.07 and average inbreeding coefficient of 0.10 in the same period (Additional file 4).

In 1995, the breed name Thai was registered and the breed was founded on animals, imported from several countries and on Siamese cats with a less extreme conformation. Over the past 20 years, the inbreeding coefficient and population mean kinship were quite low although they tended to increase. In 2016–2017, the average inbreeding coefficient and population mean kinship were equal to 0.05 and 0.03, respectively.

**Fig. 3:**
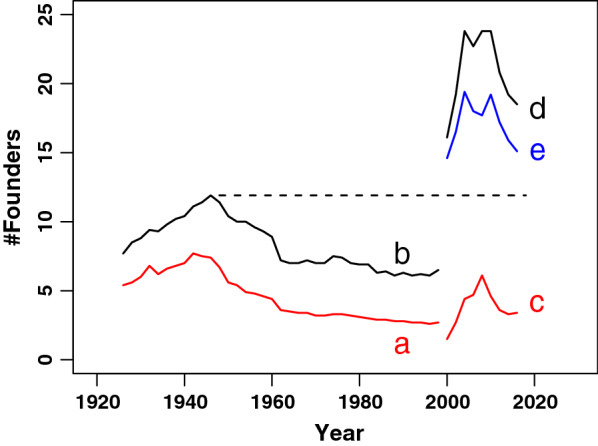
Historic gene diversity on the scale of founder genome equivalents.** a** Native genome equivalents (NGE) of SIA population (red);** b** FGE of SIA population (black);** c** NGE of the THA population (red);** d** FGE of THA population (black). The dashed line indicates the historic maximum FGE of the SIA population in 1940;** e** FGE minus NGE, mainly representing the historic contribution of SIA to THA (blue). A period of 2 years was taken as an approximate generation interval

Figure 3 shows how the genetic diversity within the Siamese and the Thai breeds has evolved on the scale of founder genome equivalents. Throughout the years, the contribution from other breeds except for Oriental Shorthair, to the Siamese breed has been relatively low, with the *NGE* and *FGE* (Trace a and trace b), reaching 4.2 and 7.0 respectively in 1998-1999. The situation is different for the Thai breed (Trace c and d). The *FGE* peaks at 23.8 in 2007 but rapidly decreases to 15.2 in 2016-2017 and the *NGE* shows a maximum of 5.4 around 2009, and drops to 3.1 in 2016-2017. Considering the Thai as a distinct breed, it has received a significant contribution from the Siamese cats with moderate conformation during the early years when the breed was founded. The difference between the *FGE* and *NGE* within the Thai breed (Trace e) represents the known contribution of Siamese cats to the Thai breed. It runs slightly above the *FGE* level within the Siamese breed between 1940 and 1960. If one considers Thai cats as a subpopulation of the Siamese breed, the Thai *NGE* could reflect the yet unknown lineage from the original Siamese population in Europe. The Thai *NGE* of the current population dropped by 9.7 units compared to the Siamese *NGE* in 1946, i.e. when it reached a maximum of 12.8 and before the modern Siamese was developed.

### Cluster analysis

A preliminary pilot study, which we conducted in 2016, on a selection of 124 Dutch individuals, showed a lower inbreeding coefficient (${\overline{F}}$= 0.01) and a low population mean kinship (${\overline{mk}}$= 0.03). Knowing that breeders outside the Netherlands traditionally apply line breeding more often, the increased inbreeding values in the current European population as shown in Fig. 2 are probably caused by regional differences. Since the individuals from different regions are not related to each other, on average, these animals contribute to a low mean kinship and a higher coefficient of inbreeding when considered together with individuals from other regions.

**Fig. 4:**
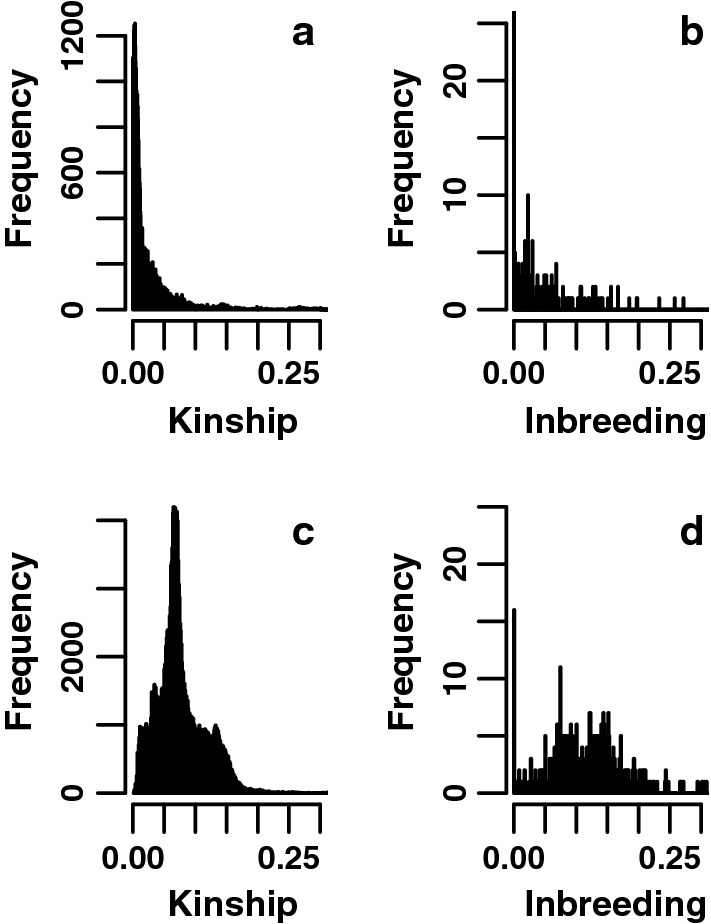
Distribution of kinship values in the current Thai and Siamese populations. **a** Distribution of pairwise kinship values across all the parents of the Thai kittens, born in 2016–2017 (N=200). The histogram represents the off-diagonal elements of the kinship matrix, i.e. of all kinship coefficients between different individuals and covers values between 0 and 0.55 in 0.001 intervals. The first datapoint at zero kinship was removed to obtain a clearer overview on the data. **b** Distribution of inbreeding values across the same individuals. The coefficient of inbreeding is calculated from the diagonal elements of the kinship matrix. **c** Distribution of pairwise kinship values across all the parents of the Siamese kittens, born in 1998-1999 (N=462). The first datapoint at zero kinship was removed to obtain a clearer overview on the data. **d** Distribution of inbreeding values across the same individuals

Regional differences between families were further explored by plotting all pairwise kinship coefficients between different individuals, i.e. by discarding the diagonal elements from the kinship matrix, which represents the kinship between an individual with itself. Since subsequent generations overlap considerably, we used age groups of two subsequent years to have an approximate representation of the generation interval of the current breeding animals (Additional file 5). Figure 4a shows the results for the current Thai population of 200 breeding animals, which are the parents of the kittens born in 2016 or 2017. This plot shows a skewed distribution with the highest peak at the lowest kinship values. The occurrence of several peaks just above zero, around 0.04 and 0.07 and possibly a smaller one around 0.13 confirm that subpopulations can be differentiated. The same pattern is observed for the coefficient of inbreeding (Fig. 4b). The kinship and inbreeding distribution with the highest peak at zero indicate a population in which outcrossing is the predominant breeding strategy.

The pairwise kinship coefficients and coefficients of inbreeding for the Siamese breed show a different pattern (Fig. 4c, d). In this case, the 462 parents of the kittens that were born in 1998 or 1999 were selected. This group may also contain parents of the newly registered Thai breed. These graphs show strong peaks, centered around higher kinship or inbreeding values, and only small contributions from unrelated individuals. Such patterns indicate that inbreeding or line breeding strategies were applied.

**Fig. 5:**
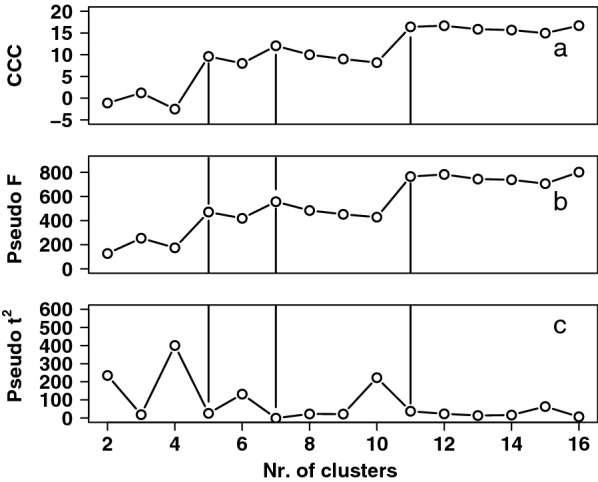
Determining the optimal number of hierarchical clusters. **a** The cubic clustering criterion (CCC); **b** The pseudo-F criterion; **c** The pseudo-$\mathrm {t^2}$ criterion for assessing the optimal number of hierarchical (UPGMA) clusters within the Thai cat population (2016–2017). Traces are evaluated from right to left. Optimal number of clusters are found at the top of a rising edge (CCC, pseudo-F) or at the bottom of the right flank of a peak (pseudo-$\mathrm {t^2}$). From these plots, the optimal number of clusters is considered to be 5 (largest discontinuity)

**Fig. 6:**
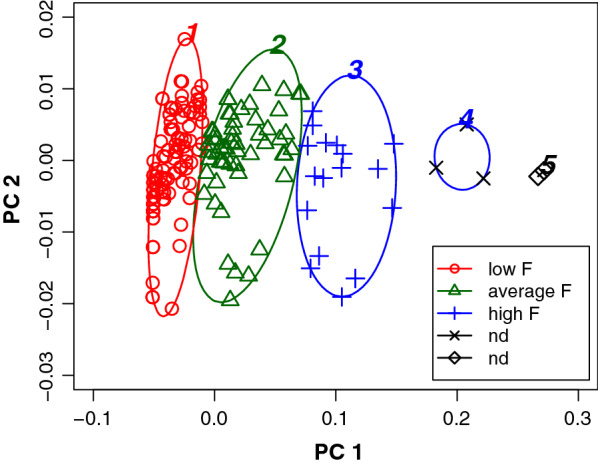
Classification of subpopulations using hierarchical cluster analysis. Hierarchical clustering separates the Thai cat population (200 individuals in 2016–2017) into three subpopulations. The solution is visualised in a PCA plot along the two principal component axes PC 1 and PC 2, with PC 1 being largely determined by the inbreeding coefficient. The clusplot program applies automatic scaling of the axes. The level of inbreeding is indicated in the legend. Color cycling inadvertently plots ellipse 4 in blue

For a more detailed analysis, Ubbink [25] proposed hierarchical cluster analysis using the kinship matrix as input. A variation of this approach was used by Oliehoek [26] to investigate the relations between Icelandic Sheepdogs in different countries. In our study, we applied the latter approach by using clustering criteria to determine the optimal number of final clusters and by including all individuals back to the founder population in the calculation of the kinship matrix. However, we explored the use of the individual mean kinship and inbreeding coefficients as input to the cluster analyses (Additional file 6). Various clustering criteria indicated that dividing the dataset of 200 Thai individuals into five clusters should provide the best solution (Fig. 5), although clusters 4 and 5 only consisted of two and three individuals, respectively. The latter two were ignored in the subsequent analysis. Recalculating mean kinship and inbreeding coefficients for the separate clusters indicated that most breeding animals were included in a single, large cluster of 119 individuals with an average mean kinship of about 0.03 and a low inbreeding coefficient of about 0.02 (Table 1). A few other breeding animals were put in cluster 2, with a mean kinship of 0.04 but a higher coefficient of inbreeding (${\overline{F}}$= 0.07). The other cluster represented animals with an average mean kinship but a high average inbreeding coefficient of 0.15. Figure 6 shows the classification of the individuals into separate subpopulations. The separation is mostly based on the differences in inbreeding coefficient (Table 1), which is represented by the first principal component along the PC 1 axis.

**Table 1:** Comparison between hierarchical and model-based clustering of current Thai population (parents of kittens, born in 2016–2017)

Cluster	UPGMA									Model-based								
	N	ECG	${N_{e}}$	${\overline{mk}}$	${\overline{F}}$	FGE	${N_{oc}}$	NGE	${N_{ocatN}}$	N	ECG	${N_{e}}$	${\overline{mk}}$	${\overline{F}}$	FGE	${N_{oc}}$	NGE	${N_{ocatN}}$
1	119	11.4	103	0.03	0.02	16.7	23.3	4.2	4.5	26	7.3	25	0.04	0.00	12.5	13.7	3.8	2.5
2	58	11.5	57	0.04	0.07	12.5	14.1	1.4	2.1	84	12.9	66	0.04	0.02	12.5	15.1	3.3	3.5
3	18	10.9	17	0.04	0.15	12.5	7.5	2.9	1.8	32	10.4	30	0.04	0.13	12.5	13.9	2.9	2.5
4	2	nd								58	11.6	57	0.05	0.07	10.0	11.4	1.3	1.7
5	3	nd																
total	200	11.4	191	0.03	0.05	16.7	26.7	3.6	5.6	200	11.4	191	0.03	0.05	16.7	26.7	3.6	5.6

The small hierarchical clusters 4 and 5 were excluded from further analysis

**Fig. 7:**
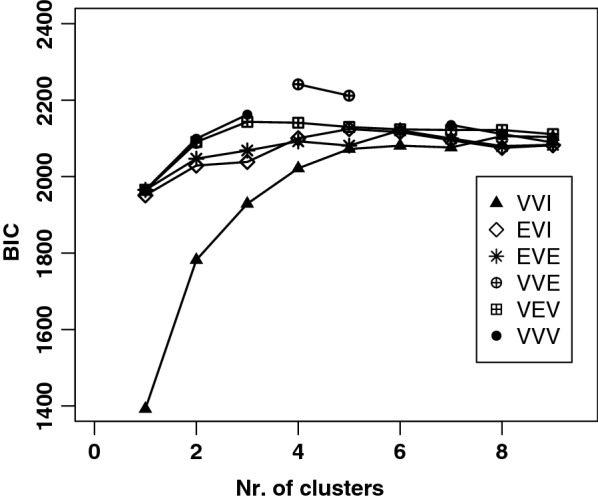
Determination of the optimal number of model-based clusters. The Bayesian Information Criterion (BIC) is used to determine the optimal number of model-based clusters within the Thai cat population (2016–2017). The model type and number of clusters are found where the BIC value is maximal. The maximum value is reached when using the VVE model and four clusters

Since the hierarchical clustering approach did not provide a clear separation between classes of animals, as an alternative, we performed a model-based cluster analysis of the same data, as developed by Fraley [22]. Evaluation of the BIC for up to nine clusters, showed that an optimum was found at four clusters, using the VVE model (variable volume, variable shape, equal orientation. Fig. 7). Using this approach, the distribution of the animals across the clusters is markedly different.

**Fig. 8:**
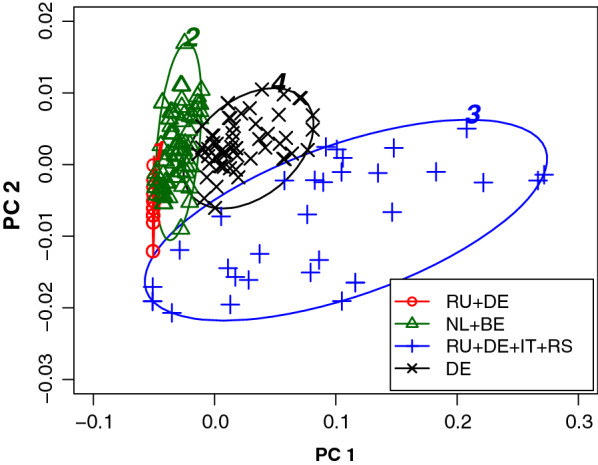
Classification of subpopulations using model-based cluster analysis. Model-based clustering separates the Thai cat population (200 individuals in 2016–2017) into four subpopulations according to a different algorithm. The solution is visualised in a PCA plot along the two principal component axes PC 1 and PC 2, with PC 1 being largely determined by the inbreeding coefficient. The clusplot program applies automatic scaling of the axes. Major regions of origin are indicated in the legend

After having identified the animals in the separate clusters, mean kinship and inbreeding coefficients were recalculated for each cluster. These results are summarised in the right part of Table 1. The first cluster is mainly populated with Russian animals, including those living in The Netherlands, plus a few German and Italian animals. The second cluster consists mainly of animals of Dutch and Belgian origin but also contains a few German animals. The third cluster is mainly populated with Russian and German animals with some additions from Serbia and Italy. Finally, the fourth cluster is mainly populated with German animals. Remarkably, clusters 1, 2 and 3 have the same values for ${\overline{mk}}$ although the animals are known to be completely unrelated. The third cluster is characterised by a relatively high ${\overline{F}}$ of 0.13. Furthermore, the first cluster shows a reduced number of equivalent complete generations compared to the other clusters. These results are visualised in Fig. 8, which shows the alternative classification of the individuals into subpopulations. The region of origin of the majority of individuals in each subpopulation is indicated in the legend of Fig. 8. Cluster 3 stands out because of the large variation along both principal component axes, which represent inbreeding coefficient and mean kinship, respectively. In summary, compared to hierarchical clustering, model-based clustering can detect regional subpopulations and distributes information on potential breeding animals across more clusters when applied to the current dataset.

Although historically, Siamese cats have contributed to the Thai breed, it is remarkable that the Thai cats have a low population mean kinship compared to the Siamese population. As already suggested by the results in Figs. 2 and  4, the existence of a Siamese subpopulation with a lower mean kinship compared to the entire population, could explain this difference. In order to investigate the relation between the Thai and the Siamese breeds, we performed cluster analysis on the 462 parents of the Siamese cats, born in 1998 or 1999. Allegedly, this cohort contains possible ancestors of the later Thai population (Additional file 7). For the Siamese breed of this age group, seven hierarchical or six model-based clusters (VEV model: variable volume, equal shape, variable orientation) were found to give an optimal division. The seventh hierarchical cluster was ignored since it contained only two individuals. The comparison between hierarchical and model-based clustering is shown in Table 2.

**Table 2:** Comparison between hierarchical and model-based clustering of the Siamese cat population (parents of kittens, born in 1998–1999)

Cluster	UPGMA									Model-based								
	N	ECG	${N_{e}}$	${\overline{mk}}$	${\overline{F}}$	FGE	${N_{oc}}$	NGE	${N_{ocatN}}$	N	ECG	${N_{e}}$	${\overline{mk}}$	${\overline{F}}$	FGE	${N_{oc}}$	NGE	${N_{ocatN}}$
1	22	10.8	22	0.03	0.00	16.7	14.9	3.3	2.2	112	18.6	111	0.05	0.05	10.0	18.0	2.9	3.0
2	177	21.7	174	0.07	0.07	7.1	11.9	2.9	3.4	39	17.9	38	0.05	0.22	10.0	11.5	3.1	3.0
3	35	23.5	34	0.10	0.21	5.0	5.8	2.5	2.4	62	23.3	62	0.09	0.13	5.6	6.1	2.8	2.7
4	12	20.8	11	0.10	0.31	5.0	4.9	3.1	2.0	72	23.4	70	0.10	0.09	5.0	5.9	2.5	2.6
5	209	24.4	206	0.12	0.14	4.2	6.1	2.3	2.7	127	24.8	125	0.14	0.14	3.6	3.9	2.0	2.1
6	5	6.8	4	0.14	0.14	3.6	3.6	1.7	0.7	50	25.2	49	0.16	0.19	3.1	3.2	1.9	1.9
7	2	nd																
Total	462	22.3	461	0.08	0.12	6.2	21.2	2.6	3.4	462	22.3	461	0.08	0.12	6.2	21.2	2.6	3.4

From these results, the first hierarchical cluster stands out with a low mean kinship and zero inbreeding coefficient, but also a lower number of equivalent complete generations, compared to the other large clusters. The second hierarchical cluster has an average mean kinship (${\overline{mk}}$=0.07) and a somewhat lower average inbreeding coefficient (${\overline{F}}$=0.07). The other clusters have a high mean kinship combined with a high inbreeding coefficient. The first model-based cluster is characterised by a relatively low mean kinship (${\overline{mk}}$=0.05) as well as a low inbreeding coefficient (${\overline{F}}$=0.05), combined with a high number of equivalent complete generations (ECG=18.6). When tracing the names of the animals in the clusters, known ancestors of Thai cats can be found, almost exclusively, in the second hierarchical cluster or the first model-based cluster.

### Optimal contribution selection

Calculating optimal contributions is a way to minimise the population mean kinship and thereby provides the possibility to maximise the genetic diversity within a population. Simultaneously, this also minimises the level of inbreeding. The results of these calculations, presented in units of founder genome equivalents, are listed per cluster in the rightmost four columns of Table 1 (Thai, year group 2016–2017) and Table 2 (Siamese, year group 1998-1999). When considering the entire European Thai population, the *FGE* can be increased from 16.7 to achieve a potential diversity of 26.7. However, the gain within the individual clusters is less pronounced. The first, large hierarchical cluster can reach 23.3 and the second hierarchical cluster can be increased up to an ${N_{oc}}$ of 14.1. The second and fourth model-based clusters, which contain mostly Dutch and German animals, can increase the *FGE* from 12.5 to 15.1 and from 10.0 to 11.4, respectively. For all UPGMA and model-based clusters, the ${N_{ocatN}}$ is practically not higher than the *NGE* or optimisation is not successful.

The overall genetic diversity of the Siamese population, given in founder genome equivalents, could be increased from 6.2 to 21.2 but only the second and fifth hierarchical clusters show a possible gain from 7.1 to 11.9 and 4.2 to 6.1 (Table 2, left). When dividing the Siamese population in model-based clusters Table 2, right), the first subpopulation of 112 animals can significantly increase the *FGE* to obtain a potential diversity of 18.0. The expected gain within the other clusters is rather limited.

## Discussion

### Comparison with other breeds

Currently, there are only a few population genetic studies on cat breeds available and those, based on pedigree data, do not include any of the oriental breeds.

Mucha [27] studied six relatively popular breeds (British Shorthair, Maine Coon, Norwegian Forest Cat, Persian and Exotic Shorthair, Russian Blue and Siberian) in Poland over a period of 25 years and found average coefficients of inbreeding of 1.7–3.8 %. Leroy [28] analysed five larger breeds (Bengal, Birman, Chartreux, Devon Rex and Maine Coon) and three breed groups (Abyssinian and Somali, British Shorthair, British Longhair plus variants and Persian and Exotic Shorthair) in France between 2003 and 2010 and found similar values for inbreeding and mean kinship coefficients or derived diversity measures. These results seem to underestimate kinship since data were available omly over a limited time interval. As is often the case, common ancestors, which contribute to an increased kinship value, do not show up on a standard pedigree of four or five generations, issued by registries.

Using marker-based DNA techniques, Menotti-Raymond [29] found an expected heterozygosity (unbiased gene diversity) ${H_{e}}$ of 0.52-0.62 and a within-population inbreeding, ${F_{is}}$, of 0.10 for the Siamese breed and ${H_{e}}$ of 0.72 and ${F_{is}}$ of 0.19 for the Oriental Shorthair. Since this approach focusses on a number of short DNA sequences selected at particular loci, rather than on the pedigree, this approach systematically reports lower estimates for diversity measures. Although ${F_{is}}$ for the Siamese corresponds with our ${\overline{F}}$ averaged across the entire European Siamese population in 1998–1999, a regional bias cannot be excluded.

Our study reveals the particular history of the Thai or traditional Siamese cat breed, which has a conformation similar to that of the original Siamese cat before 1960 that descends from the animals imported from Siam in the early 20th century. The high number of equivalent complete generations and the oldest traceable ancestor of the current population underpin the relation between the Thai and Siamese breeds. The population mean kinship of the current population on the Northwestern European mainland is 0.03 and the average coefficient of inbreeding is 0.05. However, the Siamese breed has developed differently, with a relatively high population mean kinship of 0.08 and a high average coefficient of inbreeding of 0.12, the latter corresponding approximately with the data from the sample of 32 Siamese cats worldwide [30] and the sample of 35 cats from the North American continent [29].

### Contributions by other breeds

Since conservation of the native gene pool of the original Siamese is a major objective, minimisation of contributions by other breeds is an issue when selecting breeding animals. However, this rapidly becomes a complicated topic since from 1995 the Siamese and Thai are considered as separate breeds, although genotypically, both breeds are very much alike.

Using the optiSel package, it is possible to calculate the genetic contribution from other breeds to the current Thai cat population. Only Siamese and, to a lesser extent, the Oriental Shorthair have contributed to the Thai breed. Many of the current breeding animals have genetic contributions of about 0.5 from Siamese cats (Additional file 8). Based on the difference between the *FGE* and the *NGE*, Fig. 3 shows that during the founding phase of the Thai breed, about 18 founder genome equivalents or 76 % were captured through this relationship. Cluster analysis has demonstrated that a part of this contribution comes from a Siamese subpopulation that is not related to most of the Siamese population. This historic contribution is unavoidable and minimisation is not necessary since this actually links the original Siamese cats with the current Thai cats through a selected subpopulation with a less extreme conformation, coupled to a low population mean kinship (Table 2).

Currently, it remains difficult to trace the origin of the other animals, corresponding to about 3.4 founder genome equivalents at native alleles, which were used to build the European Thai cat population, as no pedigree information was available. Molecular biological techniques may provide an answer to this question. By analysing microsatellite marker data using Bayesian cluster analysis, Lipinski and coworkers [30] identified four clusters of cats which could be related to Europe, the Mediterranean basin, East Africa and Asia as the region of origin. Moreover, the cats in various Asian regions have developed in relative isolation. This means that throughout the years, the current cat population in Thailand has not received any contribution from other breeds. Using the same approach would provide an insight into the extent of the relationship between the current European Thai cat population and the cats that live in Thailand and are descendants of the same ancestors which provided the founders of the European population about 100 years ago.

In an effort to introduce e.g. new coat color patterns, breeders occasionally perform outcrosses of Thai and contemporary Siamese and this might be regarded as real introgression of a foreign breed. Outcrosses as outlined in this example should at least be performed with care since with the introduction of foreign genes from a more strongly inbred population, there is an increased risk of simultaneously introducing deleterious alleles, that occur at high frequencies but are not present in the current population. It should be noted that some registries e.g. the FIFe does not allow such outcrosses as part of the recognition of the Thai as a separate breed [31]. DNA testing for genetic defects that are known to be present within the Siamese breed should at least be considered. Another option could be to concentrate breeding activities on the four traditional coat colors i.e. seal point, chocolate point, blue point and lilac point, such that there is less need to include individuals from other breeds.

Another aspect of genetic contributions by foreign breeds within cat populations is the fact that several breeds are defined by only a few genetic traits. For instance, it is quite frequent that two green-eyed Oriental Shorthair cats that are heterozygous for colour, produce pointed kittens with blue eyes and solid-coloured kittens with green eyes in the same litter. The blue-eyed kittens are surprisingly registered as Siamese although both parents are Oriental Shorthairs. In this case, the software corrects the breed of the offspring to Oriental Shorthair, resulting in incorrect breed information. In particular the kinship at native alleles, which relies on a specific breed, is distorted by this feature. Since we wish to focus on the Thai breed, this was not investigated in more detail.

### Maintaining optimal genetic diversity

Currently in The Netherlands, Thai breeding animals are selected such that they have a zero coefficient of inbreeding across the last four generations and, if possible, no common ancestors over ten generations. Another criterion is an ancestor loss coefficient (AVK) of 85 % (i.e. a maximum loss of 15 %). These parameters are readily provided by commercial animal keeping software packages. Recently, it has become clear that this policy is difficult to maintain, mainly because there is only a limited number of sires available. The analyses based on kinship, presented in this report, provide alternative ways to maintain or improve genetic diversity within the European Thai cat population. In conservation breeding, achieving an *FGE* of 20 or more is considered adequate for a population to maintain its own gene diversity [32]. As the overall results of the combined subpopulations suggest, the gene pool seems sufficiently large to achieve this objective. When focusing on the Dutch and German subpopulations, given by the model-based clusters 2 and 4 (Table 1, right), it is clear that these populations do not meet this criterion when relying on optimal contribution selection (OCS) alone. Moreover, an optimum as proposed by OCS is difficult to reach, since there is no central authority, that supervises the breeding program. Rather than keeping certain lines private, which is common practice within the Cat Fancy organisation, breeders from different clusters should be encouraged to collaborate by providing stud services or by mutually exchanging breeding animals. Since animals from different clusters have little or no relationships, both subpopulations should benefit from such initiatives. As pointed out by Caballero [18], breaking up a strict division into subpopulations combined with minimisation of kinship, reduces the overall ${\overline{F}}$ and consequentially reduces the risk of inbreeding depression. Another measure into which the breeders in the second (Dutch) cluster should invest is to incorporate more males to obtain a more balanced male to female ratio, i.e. a higher effective population size (${N_{e}}$). In this situation, maximisation of ${N_{e}}$ minimises the rate of increase of average mean kinship, which is proportional to $1/(2*N_e)$ [18]. A possible outcome is illustrated by the results of the hierarchical cluster 1 (Table 1, left). By recombining selected animals from all model-based clusters, a potential diversity of 23.3 can be obtained. Currently, this group of 119 animals has the same, low population mean kinship as the entire population of 200 animals.

Although in 1998−1999, the genetic status of the Siamese population was less favorable, the genetic diversity of the first model-based subpopulation, consisting of 112 animals could be improved from 10.0 to a potential diversity of 18.0 founder genome equivalents by applying OCS (Table 2, right). It seems unlikely that breeders of modern Siamese cats will seek to collaborate with breeders of animals with a less extreme conformation to achieve an even higher genetic diversity, since this would imply a deviation from the current breed standard.

## Conclusion

Using cluster analysis, we have shown that the current Thai or traditional Siamese cat population, living on the European mainland, is linked to a subpopulation of Siamese cats, that can be related to the founder animals, originally imported from Siam in the early 20th century. Although related, the Thai has a much lower population mean kinship (${\overline{mk}}$=0.03) and a lower average inbreeding coefficient (${\overline{F}}$=0.05), compared to the Siamese cat population (${\overline{mk}}$=0.08 and ${\overline{F}}$=0.12, respectively). This difference can be attributed to the fact that the Thai breed was built by combining selected Siamese cats with a less extreme conformation and a relatively low mean kinship with founders, registered as Thai. Furthermore, the breeding strategy that is followed, is based on mating less related animals (outcrossing within the breed) rather than adhering to line breeding, and thus the Thai breed does not show any signs of inbreeding depression. With model-based cluster analysis, it was possible to separate the Thai population in regional subpopulations, each having distinct values for ${\overline{mk}}$ and ${\overline{F}}$. By strictly applying optimal contribution selection, to obtain a minimal inbreeding coefficient, only a limited increase in genetic diversity within the regional subpopulations can be reached. However, in the absence of a supervised breeding program, this objective will not be reached in practice. By combining breeding animals from various regions into a single cluster, as illustrated by the first hierarchical (UPGMA) cluster, which contains 119 animals (Table 1), already a potential diversity of 23.3 up to 26.7 founder genome equivalents can be reached. This value meets the recommendation for a population, capable of supporting and maintaining its own genetic diversity.

## Supplementary Information

**Additional file 1.** Collected traditional Siamese pedigree data. Pseudonymized version (December 2018). Breeds are recoded using the EMS abbreviations. Unfortunately, the country of origin is registered together with the pedigree name. This information gets lost upon recoding pedigree names to numeric data.

**Additional file 2.** Completeness of Thai cat pedigrees. R script for analysing pedigree completeness per generation for males and females separately.

**Additional file 3.** Historic development of mean kinship and inbreeding. R script to calculate and plot historic kinship and inbreeding of the Thai and Siamese breeds, using. Additional file 1 as input. This produces Figs. 2 and 3. It runs overnight and requires at least 12 GByte RAM. If more resources are available, alternative optiSel functions can be used to yield a more compact code.

**Additional file 4.** Historic development of mean kinship and inbreeding. R script to calculate and plot historic kinship and inbreeding of the Oriental Shorthair breed, between 1955 and 1999, using Additional file 1 as input.

**Additional file 5.** Comparison of distribution of mean kinship and inbreeding. R script to plot the distribution of kinship and inbreeding coefficients of the Thai and Siamese breeds, using Additional file 1 as input. This produces Fig. 4.

**Additional file 6.** Cluster analysis of the Thai breed. R script for hierarchical (UPGMA) and model-based cluster analysis of the Thai breed (parents of kittens, born in 2016–2017). This produces Table 1 and Figs. 5, 6, 7 and 8.

**Additional file 7.** Cluster analysis of the Siamese breed. R script for hierarchical (UPGMA) and model-based cluster analysis of the Siamese breed (parents of kittens, born in 1998–1999), containing the ancestors of the newly registered Thai breed. This produces Table 2. The figures showing the optimal number of clusters are left out from the main text.

**Additional file 8.** Historic breed composition of the Thai breed. R script for analysing the contribution of other breeds to the Thai breed.

## Data Availability

All data generated or analysed during this study are included in this published article and its supplementary information files.
